# Cybrid Models of Pathological Cell Processes in Different Diseases

**DOI:** 10.1155/2018/4647214

**Published:** 2018-06-10

**Authors:** Margarita A. Sazonova, Vasily V. Sinyov, Anastasia I. Ryzhkova, Elena V. Galitsyna, Alexandra A. Melnichenko, Anton Y. Postnov, Alexander N. Orekhov, Igor A. Sobenin

**Affiliations:** ^1^National Medical Research Center of Cardiology, 15a, 3rd Cherepkovskaya Str., Moscow 121552, Russia; ^2^Institute of General Pathology and Pathophysiology, 8, Baltiyskaya Str., Moscow 125315, Russia; ^3^Institute for Atherosclerosis Research, Skolkovo Innovative Centre, Novaya St., Skolkovo, Moscow Region 121609, Russia

## Abstract

Modelling of pathological processes in cells is one of the most sought-after technologies of the 21st century. Using models of such processes may help to study the pathogenetic mechanisms of various diseases. The aim of the present study was to analyse the literature, dedicated to obtaining and investigating cybrid models. Besides, the possibility of modeling pathological processes in cells and treatment of different diseases using the models was evaluated. Methods of obtaining Rho0 cell cultures showed that, during their creation, mainly a standard technique, based on the use of mtDNA replication inhibitors (ethidium bromide), was applied. Cybrid lines were usually obtained by PEG fusion. Most frequently, platelets acted as donors of mitochondria. According to the analysis of the literature data, cybrid cell cultures can be modeled to study the dysfunction of the mitochondrial genome and molecular cellular pathological processes. Such models can be very promising for the development of therapeutic approaches to the treatment of various human diseases.

## 1. Introduction

Modelling of pathological processes in the cells is one of the most sought-after technologies of the 21st century. With the use of such models, it may become possible to study the pathogenetic mechanisms of various diseases. In addition, efforts to transfer genes, which do not have mutations, in human cells and tissues for the treatment of certain diseases, were made by the scientists throughout the world. Great success has been achieved in the implementation of genetic constructs, containing nuclear gene mutations associated with pathologies, into cell cultures. Afterwards, such structures were embedded into the nuclear genome. The final result of the transfer of genetically engineered constructs into the nuclear genome was the expression of mutant RNA in these cultures and synthesis of mutant proteins. Concerning the mitochondrial genome, such progress is not found. For example, experimental data of the members of our laboratory suggest that the transduction of genetic constructs or synthetic RNA in the mitochondria of monocytic origin cell line THP-1 was not possible. Scientists began to pay more and more attention to cybrid cultures that are more suitable models for the detection of pathological mechanisms and the development of approaches to the treatment of mitochondrial cytopathies and also other diseases associated with mutations in mtDNA.

Cytoplasmatic hybrids (cybrids) are created by fusion of mitochondria-deficient cell lines (Rho0) and cell donors of mitochondria. Since in the mitochondria with deactivated mtDNA, any fission processes do not occur because of the dysfunction of these organelles, the cell gradually loses them. Therefore, some researchers conventionally call Rho0 cells mitochondria deficient.

## 2. Materials and Methods

As a material for creating cybrid cultures, most often cell line 43B [1–8] is used; more seldom HEK293 [9, 10], HeLa [11, 12], and HL60 [13] are used.

### 2.1. Methods of Obtaining Rho0 Cell Cultures

The possibility of Rho0 human cell creation is based on the use of mtDNA replication inhibitors, such as DNA intercalating dye ethidium bromide (3,8-diamino-5-ethyl-6-phenyl phenanthridinium bromide). Relatively low concentrations of ethidium bromide (0.1 to 2 *μ*g/ml) partially or completely inhibit the replication of the mitochondrial genome, but they do not affect the replication of nuclear DNA [14].

To create cell lines of mitochondria-deficient (Rho0) standard, the methodology of King and Attardi is used, which includes two stages:

1. The verification of possibility of mitochondria-deficient cell obtaining. The native cell line is cultured in a growth medium supplemented with ethidium bromide and uridine in one embodiment and with only the addition of ethidium bromide in another embodiment. In this case, the growth medium should contain glucose and pyruvate in concentration of complete medium DMEM (2500 mg/l D-glucose, 110 *μ*g/ml of sodium pyruvate).
2. Obtaining a Rho0 cell line. At this stage, taking into account the selected cultivation conditions, cell lines are placed in growth medium supplemented with ethidium bromide and uridine. Further, ethidium bromide is excluded from the growth medium. Cultivation of mitochondria-deficient cells, according to the method, is performed on a medium supplemented with uridine.

**Figure 1:**
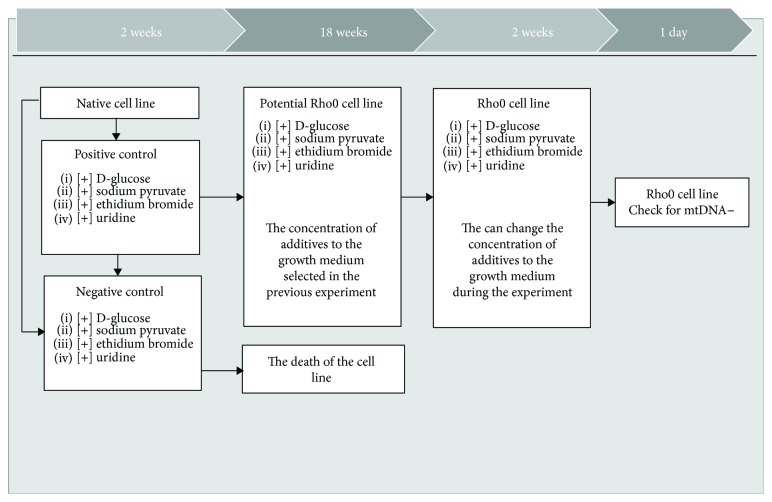
Scheme obtaining mitochondria-deficient cell lines (Rho0).

After obtaining the cell lines, stably living in previously described conditions, a cell analysis is performed in order to confirm the absence of mitochondria in these lines. The scheme of this method is shown in Figure 1.

It is noteworthy that while checking the possibility of certain cell lines to receive Rho0 cell cultures, it is necessary to choose favorable conditions of cultivation, namely, the concentration of various additives, especially ethidium bromide and uridine which may considerably vary. For example, King and Attardi for obtaining Rho0 cells from the cell line 143B.TK− cultivated the cells on a growth medium supplemented with 50 ng/ml ethidium bromide and 50 *μ*g/ml uridine [14]. Arduíno et al. for obtaining Rho0 cells from the cell line NT2 cultivated cells first with the addition of 25 ng/ml ethidium bromide and 100 *μ*g/ml uridine and after 120 days of cultivation, with increasing addition of ethidium bromide up to a concentration of 500 ng/ml within 3-4 months [15]. Mueller et al. specify that during the creation of Rho0 cells from the cell line HEK293, 500 ng/ml ethidium bromide and 100 *μ*g/ml uridine were added to the medium. Despite the assertion that the research team used the technique of King and Attardi, ethidium bromide concentration exceeded 10 times, and uridine exceeded 2 times the concentration of these additives in the original paper [10]. Therefore, in creating Rho0 cultures, an increased attention should be paid to the selection of cell cultivation conditions, particularly the concentration of ethidium bromide and uridine. Meanwhile, for cultures of various origins, the conditions of obtaining Rho0 cells can absolutely differ in both concentration of additives and in time of obtaining mitochondria-deficient cell cultures. It should be noted that these conditions may vary even for cells of the same origin but produced by different companies. In this case, a change in concentrations of ethidium bromide and uridine is required, as well as a correction of time for obtaining Rho0 culture, compared to those, which are recommended in the method.

### 2.2. Comparison of the Functional Activity of Rho0 and Native Cell Lines

Herst et al. analyzed the efficiency of the impact of all-*trans* retinoic acid and arsenic trioxide on differentiation and cell survival in human leukemic cell line HL60 and mitochondria-deficient HL60rho0. As a result, HL60rho0 cells possessed a lower differentiation ability but a higher respiratory activity than parental cell HL60. HL60rho0 cells were also significantly more resistant to apoptosis [13].

Shen et al. conducted a comparative analysis of oxygen consumption levels in cultures of human lymphoblast Molt-4 of wild type and in Rho0 cultures. The rate of oxygen consumption in mitochondria-deficient culture was significantly lower as compared to that in the native culture (7 times). The level of oxygen consumption equally changed in the native and in the mitochondria-deficient cell line Molt-4 in both the inhibition of parachloromercurobenzoate (PCMB) level and during the stimulation of sodium bisulfite by menadione (MSB). At the same time, potassium cyanide reduced and carbonyl cyanide m-chlorophenylhydrazone (CCCP) increased the oxygen consumption rate only in native culture Molt-4. The authors suggest that this can be associated with the absence of important protein subunits encoded by the mitochondrial genome, which in its turn leads to disruption of the electron transport in the respiratory chain [16].

Brar et al. compared the ultrastructural morphology of mitochondria of human epithelial cell line A549 of wild type and Rho0 cells. The mitochondria-deficient culture contained irregularly shaped mitochondria with a translucent matrix in contrast to the native cell line. In addition, the morphology of the cristae Rho0 cells was more heterogeneous: for example, both short and circular growths of the inner membrane of the mitochondria occurred. The authors analyzed the effect of bleomycin on these cell cultures, which is an antitumor agent, and in clinical use, it causes damage to the lungs, and in 1-2% of patients, it causes severe progressive pulmonary fibrosis. It was shown that Rho0-A549 cell lines are resistant to bleomycin, which, according to the authors, may be associated with the deterioration of mitochondria-dependent apoptosis [17].

Cheon et al. analyzed the role of mtDNA mutations in carcinogenesis. This research group investigated Rho0 cells obtained from cell line SK-Hep1 (liver endothelial cells). Native cell culture SK-Hep1 was used as a control culture. It was revealed that in mitochondria-deficient cells, an expression of the gene and protein level of hypoxia-inducible factor- (HIF-) 2 alpha has increased. Korean researchers suggested that mitochondrial dysfunction caused by the inhibition of mtDNA by ethidium bromide increases the angiogenic potential of tumor cells. In addition, an increase in the expression of vascular endothelial growth factor (VEGF), a key angiogenic molecule induced by HIF, was observed. Increased expression of genes associated with invasion, such as genes of urokinase-type plasminogen activator (uPA) and metalloproteinases of matrix (MMPs) in Rho0-cells of SK-Hep1 can also, according to the authors, indicate mitochondrial dysfunction, which in its turn leads to increased invasive potential of tumor cells [18].

Our research team found that Rho0 cells of THP-1 culture have lower levels of cellular and mitochondrial respiration, compared to cells of native lines of monocytic origin THP-1.

## 3. Methods of Creating Cybrid Cultures

**Figure 2:**
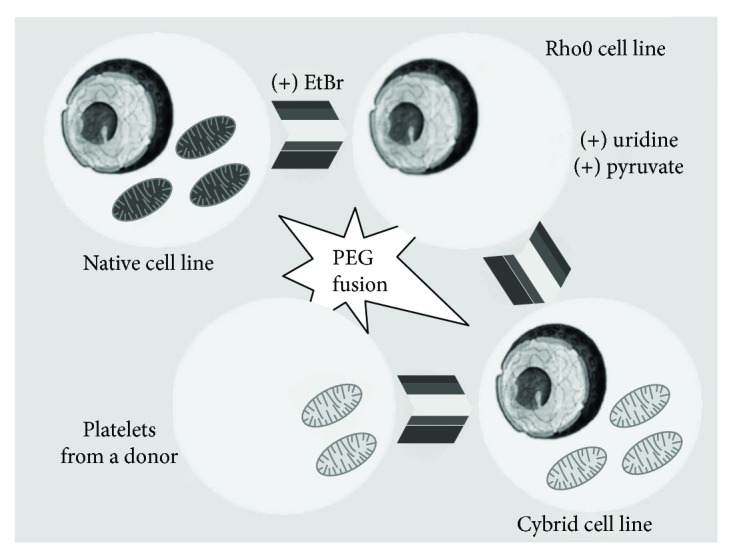
Scheme of obtaining cytoplasmic hybrids by PEG (polyethylene glycol) fusion.

There are two fundamentally different fusion methods of mitochondria-deficient lines with cell donors of mitochondria. The transfer of mitochondria into Rho0 cells can only occur during the formation of pores in the cytoplasmic membranes of cells resulting from the influence of certain chemical or physical agents on them. One of the methods involves culturing Rho0 cells with enucleated cytoplasts under the influence of a flow of direct current (electrofusion) [19]. Scientists have noted a rapid growth of cybrid cultures, observed from 20 to 28 days after the fusion. This method was used in the researches about fusion of mouse fibroblast cell line LMEB3*ρ*0 with lines BALB/cJ and C57BL/6J [20], the creation of cybrids from lymphoblasts WAL2A-Rho0 and leukocytic fraction of human blood [19], in the researches with the cultures of osteosarcoma cell 143B.TK− [21] and fibroblast LM (TK−) [22]. The second method allows the creation of cytoplasmic hybrids by merging them with platelets acting as donor cells of mitochondria [2, 3]. It should be emphasized that the use of platelet considerably simplifies the protocol of obtaining cybrid lines, since platelets contain only mitochondrial genome. Nuclear genome is absent in them. The process of creating cybrids by merging them with platelets is shown in Figure 2. Polyethylene glycol 1500 (PEG 1500) acts as a factor affecting the cytoplasmic membrane of cells. This method was used in researches on the creation of cytoplasmic hybrids based on NT2 teratocarcinoma [15], cell line 143B.TK− osteosarcoma of human [23–25] and neuroblastoma cell SH-SY5Y [26].

## 4. Cybrid Models

Cybrids are used as cellular models for studying mitochondrial dysfunction and its association with a variety of human diseases such as Parkinson's disease, Alzheimer's disease, HIV, human herpes virus type 8, MELAS syndrome, Leber's optic atrophy, and Leigh syndrome [2, 7, 8, 19, 27–29]. A significant part of the literature on this subject is works in which the association of mitochondrial genome point mutations with the emergence and the development of pathological processes, including in the cybrid cells, is studied. The results of these studies indicate a decrease in the activity of the respiratory chain complexes and oxygen consumption and reduction of ATP synthesis [2, 3, 10, 12, 29, 30].

## 5. Association of Mitochondrial Genome Haplogroups with Cell Dysfunction

Several research groups discovered an association of certain mutations and mitochondrial genome haplogroups with cell dysfunction. For example, Mueller et al. evaluated the survival index of cell line HEK293. Cybrids carrying in one case haplogroup T and haplogroup N in the other were obtained from this cell culture. During the treatment with hydrogen peroxide, cybrids with haplogroup T had a higher survival index than the other cell line [9].

## 6. The Association of Mitochondrial Genome Mutations with Syndromes

Trounce et al. found an association between mtDNA mutation m.8993T>G and dysfunction of mitochondria isolated from lymphoblasts of patients with Leigh syndrome. This association is characterized by a decrease in oxidative phosphorylation processes in the studied cells. In their investigations, a decrease in the activity to 26–50% of 3 complexes of respiratory chain and also a 30% reduction of ADP/oxygen ratio have been shown [8].

A research about creating mitochondria-deficient cell lines from cells of patients with mitochondrial encephalopathy and MERRF syndrome, having a reduced activity of cytochrome C oxidase, is rather interesting. The obtained Rho0 cells were fused with enucleate cells, HeLaCOT. As a result, cybrids with restored oxidase activity of cytochrome C were obtained [11].

## 7. Mitochondrial Dysfunction and Carcinogenesis

Some researchers study the association of mitochondrial dysfunction with oncological diseases, using various cybrid lines.

For example, Cruz-Bermúdez et al. created cytoplasmic hybrids based on cell line 143B. Their cybrids had mitochondrial genome mutations “with varying degrees of pathogenicity”: one line was with a severe pathogenicity (mutation m.8363G>A in gene tRNA-Lys) and 3 lines with a mild pathogenicity, namely, Leber's optic atrophy (LHON). The recent cell lines carried one mtDNA mutation in each cybrid culture (mutations m.3460G>A in gene MT-ND1, m.11778G>A in gene MT-ND4, and m.14484T>C in gene MT-ND6). The study results showed that these cybrid lines have mild pathogenicity and have OXPHOS dysfunction. However, they were not associated with tumorigenicity and do not cause tumors when they were used with “naked” mice [31].

Yoshii et al. determined mitochondrial dysfunction in cybrid lines based on cell line 143B carrying mutant mitochondria in patients with MELAS syndrome. In this article, radioactive Cu-diacetyl-bis (N4-methythiosemicarbazone) (Cu-ATSM) was used, which is a potential marker for the visualization of hypoxic tumors for positron emission tomography (PET). Cybrid cells with MELAS had an increased absorption of Cu-ATSM under normoxia compared to wild-type cells. It also showed that Cu-ATSM absorption correlates with the change in the level of NADH and NADPH [32].

## 8. Association of Mitochondrial Dysfunction with Alzheimer's Disease (AD) and Mild Cognitive Impairment (MCI)

At the predementia stage of Alzheimer's disease (AD) may occur MCI (mild cognitive impairment). During this stage of disease, a decrease in cognitive abilities may be observed, which often represents a transition between AD dementia and normal cognition. A group of researchers transferred mitochondria from MCI, AD, and platelets of control subjects to mtDNA-depleted SHSY5Y cells to model bioenergetic dysfunction of AD and MCI. Bioenergetics-related infrastructures and bioenergetic fluxes were characterized in the obtained cybrid cell lines. Compared to cybrids of control subjects, AD and MCI cybrids had changes in respiratory coupling, consumption of oxygen, and utilization of glucose. The level of ADP/ATP in AD and MCI cybrids was higher and the ratios of NAD1/NADH were lower [33, 34].

## 9. Possibilities of Therapy Using Cybrid Cell Cultures

Trimmer and Bennett created and analyzed cybrid neuroblastoma SH-SY5Y and NT2 with mitochondria of patients with Parkinson's disease. The research of cells in cybrid cultures revealed an increase in oxidative stress and the frequency of apoptosis compared to normal cells. SH-SY5Y and NT2 in case of applying the light therapy, characteristic for the treatment of neurological diseases, normalization in the mitochondrial respiratory activity cybrid cells SH-SY5Y and NT2 was observed [28].

In another investigation, Trimmer and colleagues studied that the possibilities of the treatment of Parkinson's disease (PD) on a cybrid model were created with the use of platelets from patients with this pathology. It should be noted that ATP, necessary for the support of axonal transport and many other cellular functions essential for the survival of neurons, is produced by mitochondrial respiratory chain. Scientists exposed cybrids to low level, near-infrared laser light and found that in these cells, mitochondrial metabolism enhanced, the level of oxidative phosphorylation increased, and redox capacity improved. The results of this investigation show the opportunity of low-level light therapy to improve defects in neuronal cells in individuals which have Parkinson's disease [35].

## 10. Conclusion

The analysis of literature dedicated to the methods of obtaining Rho0 cell cultures showed that during their creation, mainly a standard technique, based on the use of mtDNA replication inhibitors (ethidium bromide), is used. Cybrid lines are usually obtained by PEG fusion. Most frequently, platelets act as donors of mitochondria.

It should be emphasized that cybrid cell cultures can serve as models for studying the dysfunction of the mitochondrial genome and molecular cellular pathological processes. Such models can be very promising for the development of therapeutic approaches to the treatment of various human diseases.

## Abbreviations

RNA: Ribonucleic acid
DNA: Deoxyribonucleic acid
mtDNA: Mitochondrial DNA
DMEM: Dulbecco modified Eagle medium
HIV: Human immunodeficiency virus
MELAS: Mitochondrial encephalomyopathy, lactic acidosis, and stroke-like episodes
ATP: Adenosine triphosphate
ADP: Adenosine diphosphate
MERRF: Myoclonic epilepsy with ragged red fibers
OXPHOS: Oxidative phosphorylation
NADH: Nicotinamide adenine dinucleotide, reduced form
NADPH: Nicotinamide adenine dinucleotide phosphate, reduced form.
